# Mechanical analysis of a novel biodegradable zinc alloy stent based on a degradation model

**DOI:** 10.1186/s12938-019-0661-2

**Published:** 2019-04-02

**Authors:** Kun Peng, Xinyang Cui, Aike Qiao, Yongliang Mu

**Affiliations:** 1 College of Life Science and Bioengineering, Beijing University of Technology, No.100, Pingleyuan, Chaoyang District, Beijing, 100124 China; 2 Northeastern University, Shenyang, 110819 Liaoning China

**Keywords:** Biodegradable stent, Stent design, Finite element analysis, Corrosion

## Abstract

**Background:**

Biodegradable stents display insufficient scaffold performance due to their poor Young’s Modulus. In addition, the corresponding biodegradable materials harbor weakened structures during degradation processes. Consequently, such stents have not been extensively applied in clinical therapy. In this study, the scaffold performance of a patented stent and its ability to reshape damaged vessels during degradation process were evaluated.

**Methods:**

A common stent was chosen as a control to assess the mechanical behavior of the patented stent. Finite element analysis was used to simulate stent deployment into a 40% stenotic vessel. A material corrosion model involving uniform and stress corrosion was implemented within the finite element framework to update the stress state following degradation.

**Results:**

The results showed that radial recoiling ratio and mass loss ratio of the patented stent is 7.19% and 3.1%, respectively, which are definitely lower than those of the common stent with the corresponding values of 22.6% and 14.1%, respectively. Moreover, the patented stent displayed stronger scaffold performance in a corrosive environment and the plaque treated with patented stents had a larger and flatter lumen.

**Conclusion:**

Owing to its improved mechanical performance, the novel biodegradable zinc alloy stent reported here has high potential as an alternative choice in surgery.

## Introduction

Biodegradable stents provide temporary scaffolds to stenotic vessels. They can be absorbed by the human body once the remodeling of the stenotic vessel is completed [1]. These devices have great potential for decreasing risks related to long-term biological incompatibility between permanent stents and arteries, especially in case of late thrombosis [2], in-stent restenosis [3] and hypersensitivity reactions [4, 5]. However, only few biodegradable stents have been used in clinical therapy because of their poor scaffolding ability. Both the Young’s Modulus [6] and structure stability during degradation are low compared to permanent materials [7, 8] thus compromising the radial stiffness of the stent. Structural designs can be effective in improving biodegradable stents and consequently to face the related contemporary challenges.

In a previous study, we reported on a patented stent with a novel design. We confirmed that this stent has strong scaffold performance and a positive impact on the reshaping of stenotic vessels in a non-corrosive environment [9]. However, the scaffold performance in degradation conditions was not explored in that work. Indeed, the scaffold performance of biodegradable stents is extremely affected by material degradation. The structures of biodegradable stents are gradually weakened when exposed to a corrosive environment. This can even lead to mass loss if damages are severe enough. Thus, the scaffold performance of biodegradable stents is gradually decreased and eventually lost following degradation. Mechanical equilibrium between the vessel and the degraded stent evolves during the corrosion process. Thus, changes in scaffold performance significantly affect the treatment of stenotic vessels. Rapid decrease of scaffolding properties causes a severe decline of the vessel lumen size and ineffective treatment. Therefore, it is crucial to analyze the dynamics of scaffolding performance of the patented stent in a corrosive environment.

Stent degradation is a complex process simultaneously influenced by different corrosion phenomena. In previous studies, several degradation models involving uniform corrosion [10–13], stress corrosion [10, 14] and pitting corrosion [13] were reported. The corrosion mechanisms in these models were explained and changes of scaffold performance of biodegradable stents with common designs were evaluated. For example, Grogan et al. [13] developed a degradation model and predicted the corrosion effects on the mechanical integrity of bioabsorbable metallic stents. Wu et al. [14] investigated the service time of three stents with different designs. Optimized stents displayed an increase in half normalized recoil time of nearly 120% compared to common stents. Nevertheless, mechanical analyses of the stenotic vessels deployed with biodegradable stents were not performed.

Therefore, in the present study, we investigated the scaffold performance of the patented stent and its effect on reshaping stenotic vessel in a corrosive environment using finite element analysis (FEA). The patented stent and a common stent used as a control were implanted into 40% stenotic vessels. A corrosion model was subsequently applied to simulate the degradation of both stents. Radial recoiling ratio, mass loss ratio as well as von Mises stress distribution in the stents and stenotic vessels were recorded during the degradation process. It is widely established that stent geometries have strong influence on their mechanical performance. Thus, structural innovations are expected to lead to the development of high performance stents. This study will represent a significant reference for further structural designs of biodegradable stents.

## Materials and methods

### Geometry models

**Fig. 1:**
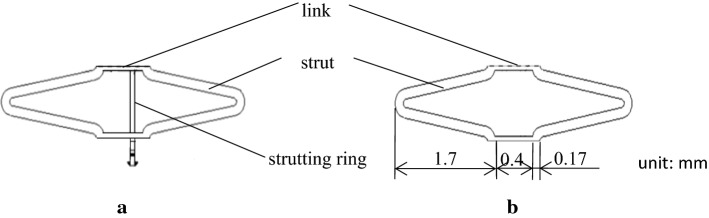
Geometries of two stent units. **a** The patented stent unit. **b** The common stent unit

**Fig. 2:**
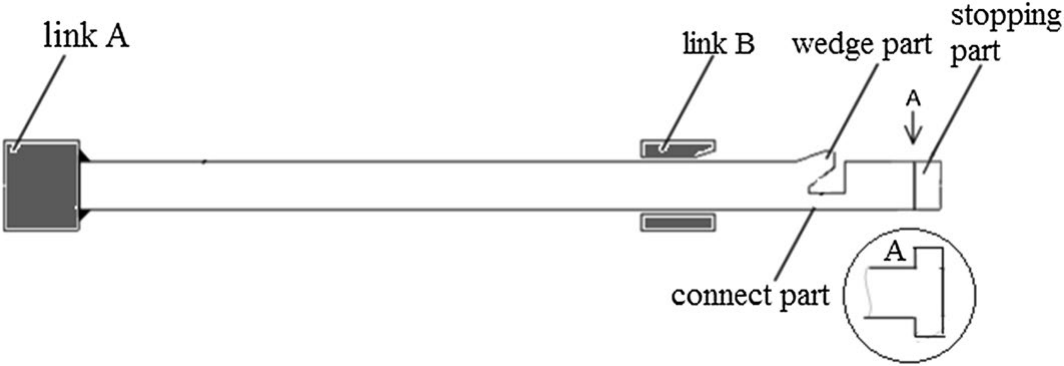
Sectional view of the short strutting ring and the links

**Fig. 3:**
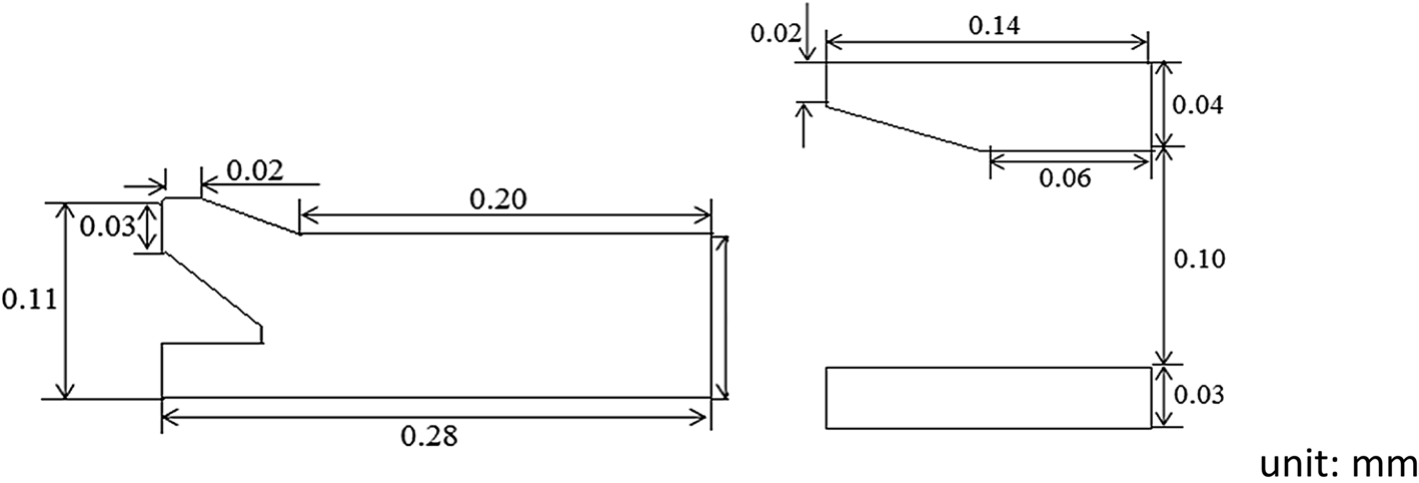
Dimensions of the short strutting ring and the link

**Fig. 4:**
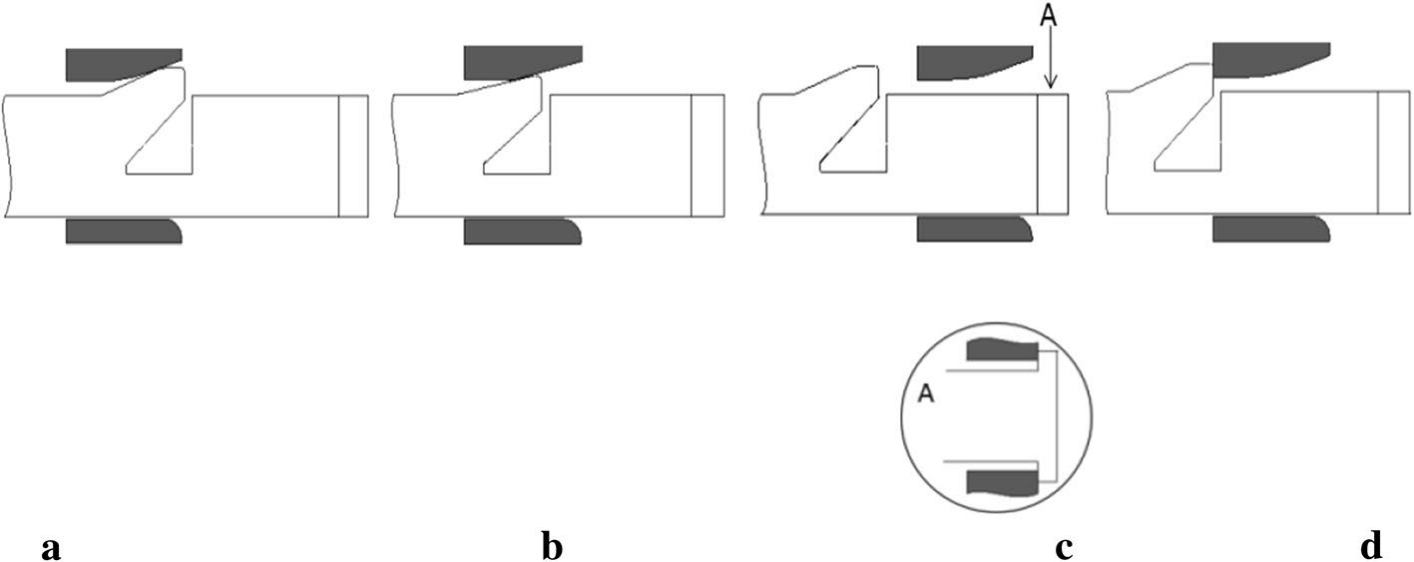
Interaction between the strutting ring and the link. **a** Initial status of the strutting ring and the link. **b** The wedge part can be compressed through the link when the strutting ring moves from right to left. **c** The wedge part recoils after through the link, while the stopping part can prevent the strutting ring from sliding out the link. **d** The interaction between the wedge part and the link can prevent the strutting ring from moving back

Figure 1a, b depict the patented stent and the common stent used as a control, respectively. Both stents harbor circumferential cycle structures and are composed of six identical units. The patented stent was designed as already described in our previous study [9]. In both the patented stent and the control, two sinusoidal struts are connected by straight links. Dimensions of the struts and the links are similar for both stents. Contrary to the control stent, each unit of the patented stent contains a short strutting ring within the link which allow the stent to expand while preventing contraction. Figure 2 illustrates details of the short strutting ring and the links. The short strutting ring consists of a wedge, a connection and a stopping part. It is tied on a solid link (link A) and runs through another link (link B). More detailed dimensions of the short strutting ring and the link are shown in Fig. 3. The cooperation between the strutting ring and the link was described in a previous work [15] (Fig. 4). The stopping part can prevent the short strutting ring from sliding out from the link.

### Material

Both stents are assumed to be fabricated with biodegradable zinc alloy. The biodegradable zinc alloy was made and tested at the Metallurgical Research Institute, Northeastern University, China. The biodegradable zinc alloy is made of zinc, magnesium and aluminum in the following proportions: Zn-3Al-1 Mg. The biodegradable zinc alloy is an elastic–plastic material and its properties are as follows: Young’s Modulus E = 74.5 GPa, Poisson’s ratio v = 0.3, yield strength = 220 MPa and ultimate strength = 325 MPa.

The mechanical behavior of vessels and plaque are highly nonlinear and these objects are assumed to be incompressible hyperelastic materials presented by a third-order Ogden and a first-order isotropic hyperelastic material model, respectively [16, 17]. The constitutive equation is described as follow:

1 $$W = \sum\limits_{i = 1}^{3} {\frac{{2\upmu_{i} }}{{\alpha_{i}^{2} }}} (\uplambda_{1}^{{\alpha_{i} }} + \uplambda_{2}^{{\alpha_{i} }} + \uplambda_{3}^{{\alpha_{i} }} - 3) + \sum\limits_{i = 1}^{3} {\frac{1}{D}}_{i} (J - 1)^{2i}$$

where *W* is the strain-energy density function. Both μ_i_ (MPa) and α_i_ are associated with the shear behaviour of materials and *D*_*i*_ describes material compressibility. The assumption of material incompressibility is realized by specifying a Poisson’s ratio of 0.49 and infinitesimal values for $D_{1} = \left( {D_{2} = D_{3} = 0} \right)$ [16]. The material coefficients are specified in Table 1 [16].

**Table 1:** Material coefficients [16]

Part	*μ*_1_/MPa	*μ*_2_/MPa	*μ*_3_/MPa	α_1_	α_2_	α_3_	*D*_1_	*D*_2_	*D*_3_
Vessel	− 1.84	1.12	0.73	21.71	22.00	21.20	4.11	0	0
Plaque	0.32	–	–	9.25	–	–	0.13	–	–

Courtesy of Holzapfel et al. and the International Journal for Numerical Methods in Biomedical Engineering

### Material degradation model

Continuum damage mechanism (CDM) illustrates the mechanical strength reduction of a material with damage accumulation [18]. The relationship between the effective stress tensor ($\sigma$) and the undamaged stress tensor ($\bar{\sigma }$) is described in Eq. (2). *D*, a damage variable, increases monotonously from 0 to 1. There is no damage in the material if $D$ is equal to 0, while *D *= 1 means that the material completely lost its properties.

2 $$\sigma = \left( {1 - {\text{D}}} \right)\bar{\sigma }$$

A biodegradable material model referring to uniform and stress corrosion was built. The global damage variable *D* is assumed to be a linear superposition of the uniform corrosion damage *D*_*U*_ and the stress corrosion damage *D*_*SC*_ (as shown in Eq. 3).

3 $$D = D_{U} + D_{{SC}}$$

The uniform corrosion damage *D*_*U*_ describes the mass loss of material when the material is exposed to aggressive environment. The damage evolution law of uniform corrosion process is supposed to be functions of $\delta_{U}$, *k*_*U*_ and *L*_*e*_ with the following formula:

4 $$\dot{D}_{U} = \frac{{\delta_{U} }}{{L_{e} }}k_{U}$$

where $\dot{D}_{U}$ means time derivative, $k_{U}$ is a parameter related to the kinetics of the uniform corrosion process and $\delta_{U}$ is a characteristic dimension of the uniform corrosion process. $L_{e}$ is the characteristic length of a finite element.

*D*_*SC*_ depicts the damage related to stress corrosion (SC) process. The damage evolution law assumed for the SC process is shown in Eq. (5) and was used by da Costa-Mattos et al. [19] to model the same phenomenon on stainless steel.

5 $$\left\{ {\begin{array}{ll} {\dot{D}_{SC} = \frac{{L_{e} }}{{\delta_{sc} }}\left( {\frac{{S\sigma_{eq}^{*} }}{1 - D}} \right)^{R} } & {\sigma_{eq}^{*} \ge \sigma_{th} > 0} \\ {\dot{D}_{SC} = 0} & {\sigma_{eq}^{*} < \sigma_{th} } \\ \end{array} } \right.$$

where $\sigma_{eq}^{*}$ is the equivalent von Mises stress, and $\sigma_{th}$ is a stress threshold under which the stress corrosion does not occur. In this model, $\sigma_{th}$ is set to 50% of the yield stress of the biodegradable zinc alloy [20]. $\delta_{SC}$ is a characteristic dimension of the stress corrosion process. *S* and *R* relate to the kinetics of the stress corrosion process and are a function of the corrosive environment. *S* and *R* are kept constant because the corrosive environment had a constant pH. Details of these relevant parameters are listed in Table 2 [14]. The remarkable table are given by Wu et al. [14].

**Table 2:** Parameters for the material degradation model [14]

$\delta_{U}$	$k_{U}$	$\delta_{SC}$	$\sigma_{th}$	*S*	*R*
0.1 mm	0.005	0.07 mm	110 MPa	0.007 mm^2^h^−0.5^/N	2

Courtesy of Wu et al. and the Materials Science & Engineering B Journal

The material degradation model was implemented into a finite element framework using the commercial code ABAQUS/Explicit 6.13 by means of a user subroutine (VUSDFLD). The stress state was calculated and updated in the explicit time integration during the whole corrosion process.

### FEA models and meshing

**Fig. 5:**
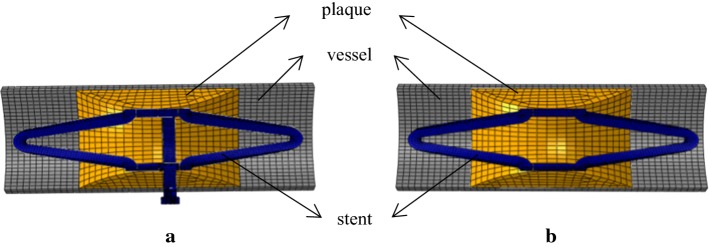
FEA models. **a** Model I. **b** Model II

As shown in Fig. 5, Model I and Model II represent the FEA models of stenotic vessels treated with the patented stent and the common reference stent, respectively. Vessel tissues in both models are illustrated by a cylinder with a length of 5 mm, an inner diameter of 4.2 mm and a wall thickness of 0.2 mm. Plaque tissues in both models have a crescent shape and are located at the middle of the vessel. Plaque tissues correspond to a maximum stenosis of 40%. The FEA models only include one-sixth of the circumferential geometries for saving computational consumption.

Hexahedral and pentahedral elements were used to mesh both models with the use of Hypermesh 13.0 (Altair, USA). The types of the hexahedral and the pentahedral elements were defined as C3D6R and C3D8R, respectively. To avoid elements distortion, elements were defined hourglass allowed in analysis. Different element sizes were chosen with regards to the geometry. Mesh sensitivity was tested by decreasing element sizes of the stents by five times. This showed that the maximum stress in the different FEA models was less than 5%.

### Boundary conditions and loads

ABAQUS/Explicit 6.13 was used for the simulations. Cyclic symmetric constraints were imposed on the corresponding symmetry nodes of both FEA models. Radial constraints were free. The whole simulation contained three simulation steps. The first two steps simulated the conventional stent implantation procedures. In step-1, both stents were expanded to the target radial displacement (0.66 mm) in stenotic vessel by exerting expansion pressure on the inner surfaces of the stents. An expansion pressure of 2.8 MPa or 2.1 MPa was used to inflate the patented stent or the common stent, respectively. Indeed, a little higher expansion pressure was applied within the patented stent to overcome the resistance caused by the interaction between the strutting ring and the link. The contact between the outer surface of the strut and the inner surface of the plaque was set to “surface-to-surface contact” and the friction coefficient was 0.1. In step-2, both stents recoiled under compression by the plaque-vessel tissues. In step-3, both stents were submitted to degradation based on the material degradation model. The degradation process was analyzed for both stents during damage evolution.

## Results

The stent radial recoiling ratio and mass loss ratio are defined by Eqs. (6) and (7), respectively.

6 $${\text{Radial recoiling ratio }} = \frac{{D_{l} - D_{s} }}{{D_{l} }} \times 100\%$$

7 $${\text{Mass loss ratio}} = \frac{{M_{loss} }}{{M_{initial} }} \times 100{\text{\% }}$$

where *D*_*l*_ is the radial expansion displacement (*D*_*l*_ = 0.66 mm), *D*_*s*_ is the radial displacement after recoiling. $M_{loss}$ is the mass loss of the stent. $M_{\text{initial}}$ is the initial mass of stent.

**Fig. 6:**
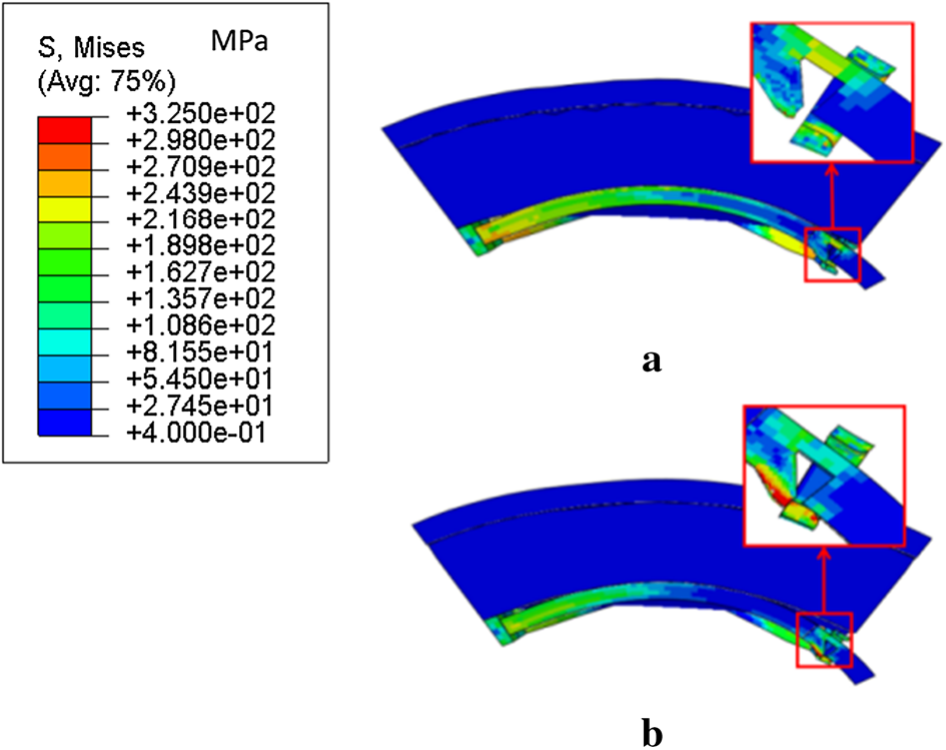
The von Mises stress distribution in Model I. **a** Step-1. **b** Step-2

**Fig. 7:**
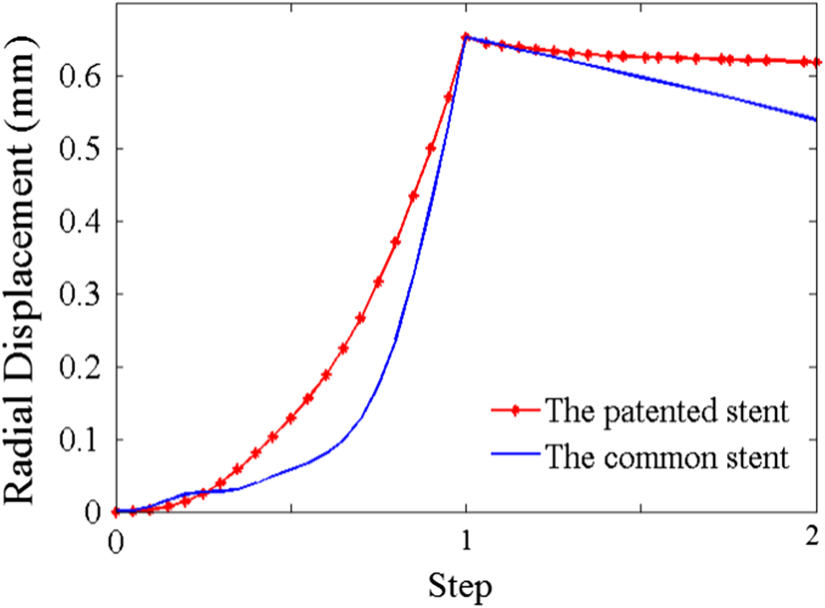
Radial displacements of the two stents during the first two steps

In step-1, the wedge part was compressed through the link and the patented stent was expanded to target displacement, as shown in Fig. 6a. In step-2, the patented stent was prevented from recoiling owing to the interaction between the wedge part and the link (Fig. 6b). The radial displacements of the stents are plotted in Fig. 7. The radial recoiling ratio of the patented and control stents were 6.2% and 18.2%, respectively. The radial recoiling ratio of the patented stent showed a marked decrease of 65.9%. In line with our previous study, the patented stent thus provided a stronger scaffold for stenotic vessel in a non-corrosive.

In step-3, the simulation time, t had almost no physical meanings because of the lack of an experimental-based identification of the corrosion parameters. However, this parameter can simply represent an evolutionary variable to allow the comparison of different designs. In order to make an effective comparison between different designs, a normalized unit time (t*) based on the longest simulation time before the fracture of the stent was chosen to illustrate the results.

**Fig. 8:**
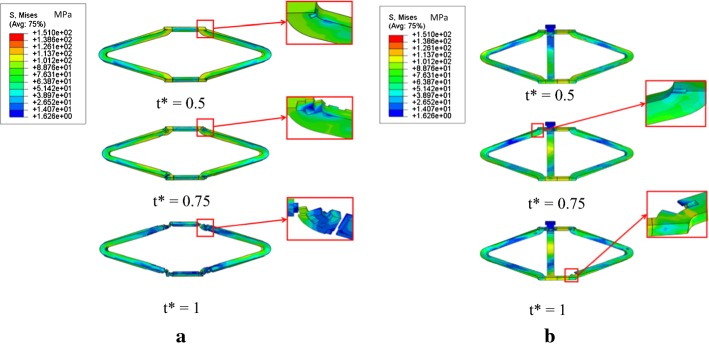
Von Mises stress distribution in the two stents during degradation. **a** Common stent. **b** Patented stent

Figure 8 depicts von Mises stress distribution in both stents during the degradation process. While degrading, materials harbored weakened structures and mass loss. Ultimately, the common stent broke down. In contrast, only few elements failed in the patented stent at t* = 1, showing that the patented stent have a longer service time. The maximum von Mises stresses of both stents at different times are listed in Table 3. During the initial period of degradation, the stents became weaker but did not loose mass. With damage accumulation, stents were gradually degraded and some thinner parts suffered from severe deformation. This explains that the maximum von Mises stress first decreased for both stents prior to increasing. The maximum von Mises stress of the patented stent located at the contact position between the strutting ring and the link. The maximum von Mises stress of the patented stent was higher compared to the common stent, indicating strong interaction between the strutting ring and the link that continuously prevented the patented stent from recoiling. Apart from the contact position, the patented stent did not suffer from extreme deformation. Therefore, the average von Mises stress only decreased from 50.71 to 39.6 MPa in the patented stent. On the other hand, that of the common stent decreased from 80.99 to 51.9 MPa during the degradation process. These data corroborated that less recoiling and failed elements occur with the patented stent.

**Table 3:** Maximum von Mises stresses at different time points

Stent	Maximum von Mises stress at different times			
	t* = 0	t* = 0.5	t* = 0.75	t* = 1
The patented stent (MPa)	325	113.8	150.4	139.3
The common stent (MPa)	271.3	121.9	116.6	129.0

**Fig. 9:**
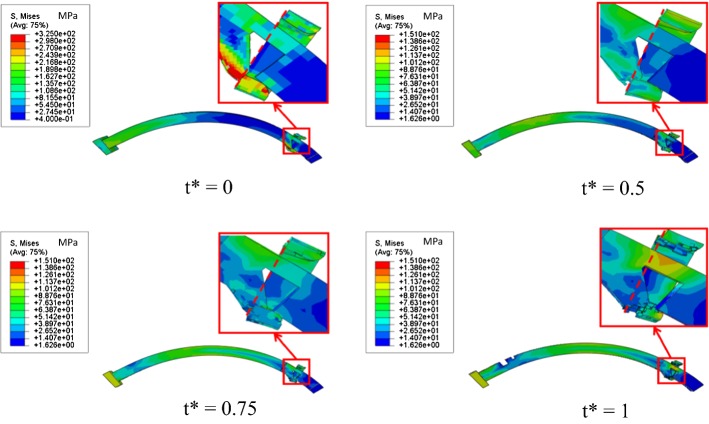
Von Mises stress distribution in the strutting ring and the link of the patented stent during degradation process

Von Mises stress distribution in a cross section of the strutting ring and the link of the patented stent are shown in Fig. 9 at t* 0, 0.5, 0.75 and 1. From t* = 0 to 1, stress corrosion attacked the touching position between the wedge part and the link B because of high stress concentration. Suffering stress corrosion, the strutting ring gradually moved back and the interaction between the strutting ring and the link was updated, as depicted by the relative position between the wedge part and the red dash line. According to the high stress region located at the wedge part and the link B, the updated interaction worked continuously to prevent the patented stent from recoiling.

**Fig. 10:**
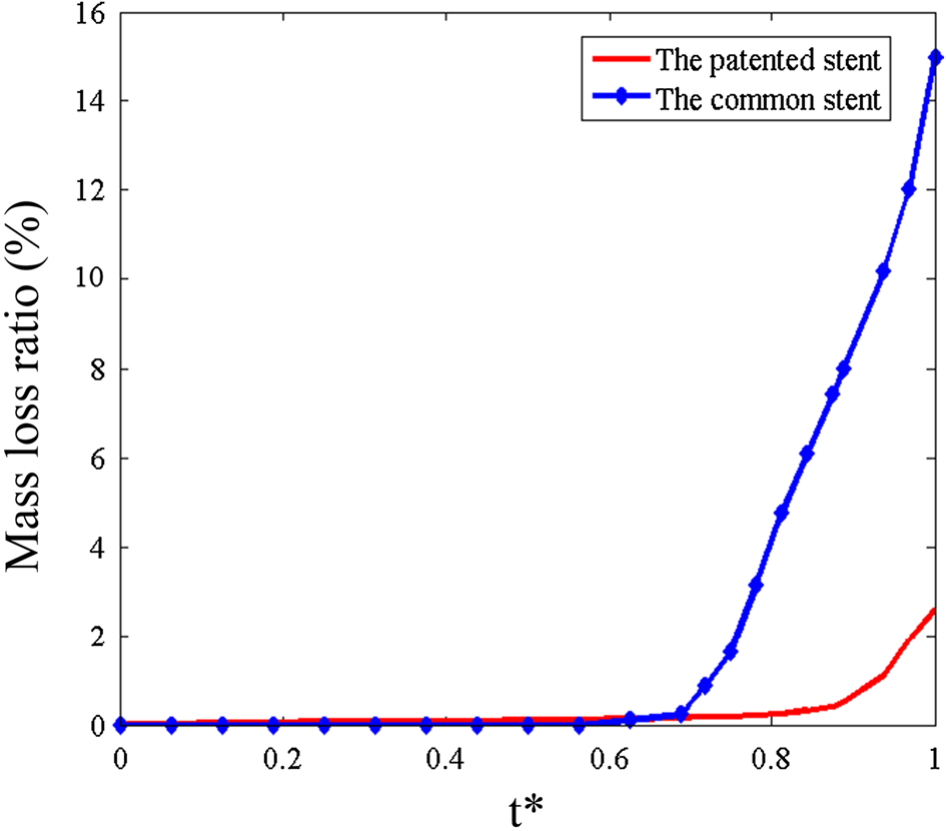
Mass loss ratio of the two stents in degradation process

**Fig. 11:**
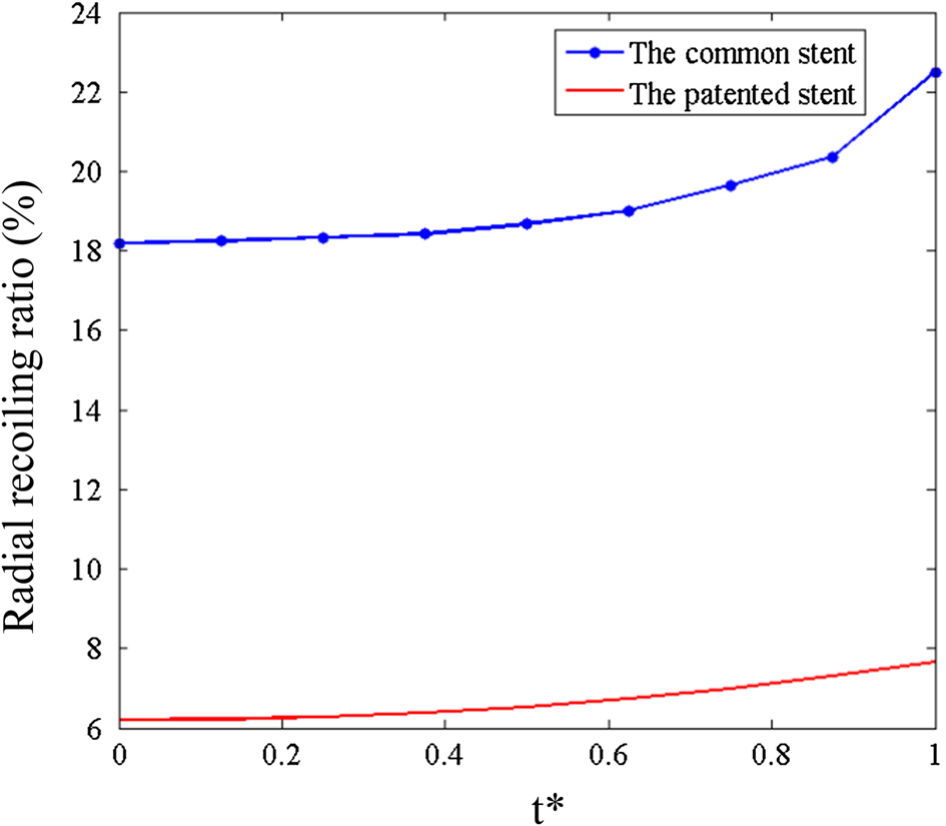
Radial recoiling ratio of the two stent in degradation process

Figures 10 and 11 show the mass loss and radial recoiling ratios of both stents. It is established that an element will be deleted once the accumulated damage (D) reaches a threshold value of 0.9. Below this threshold, the accumulated damage reduces the stiffness of materials. The radial recoiling ratio of both stents slowly increased during the initial period while the mass loss ratio was almost 0. The mass loss ratio of the patented stent (3.1%) was finally lower than that of the common stent (14.1%). The radial recoiling ratio of the common stent increased from 18.2 to 22.6% during degradation, while that of the patented stent increased from 6.2 to 7.19%. This mild increase indicates a lower rate of scaffold performance loss in corrosive environment.

**Fig. 12:**
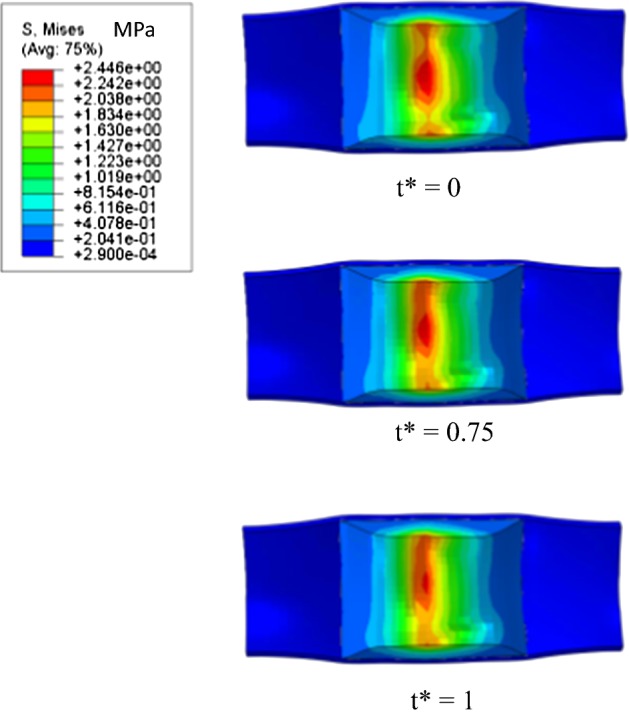
Von Mises stress distribution in plaque-vessel systems of Model I

**Fig. 13:**
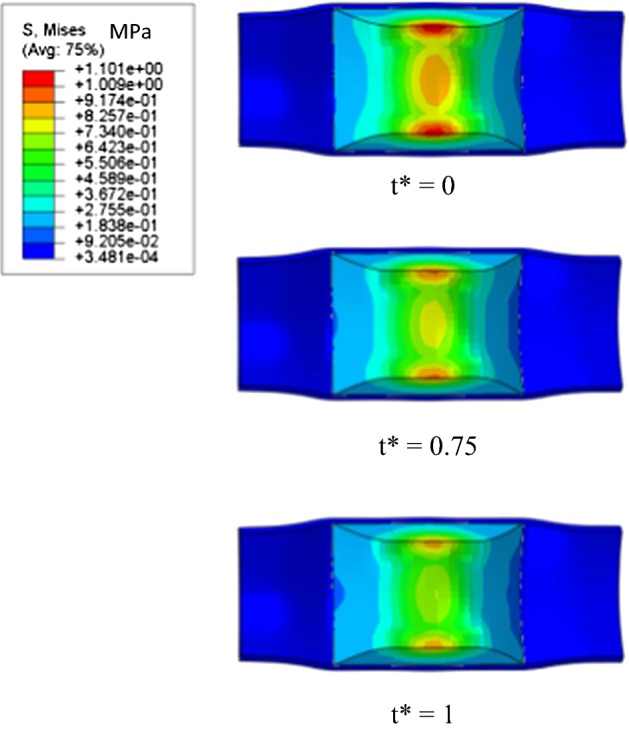
Von Mises stress distribution in plaque-vessel systems of Model II

Figures 12 and 13 show the stress distribution in the plaque-vessel tissue of Model I and Model II, respectively, when t* is equal to 0, 0.75 and 1. Plaque-vessel systems are hyperelastic and resist to deformation, thus supplying radial force to the stents. This theory held the stress distribution in plaque-vessel system of both models. From t* = 0 to 1, the maximum stress in plaques of the two models decreased with plaque recoiling. When t* = 1, the maximum stress in the plaque-vessel system of Model I (2.03 MPa) was definitely higher than that of Model II (0.85 MPa). This demonstrates that the stenotic vessel treated with the patented stent recoils less than that treated with the common stent in a corrosive environment. Therefore, the patented stent also had a positive effect on reshaping stenotic vessel during degradation.

## Discussion

In the present study, we investigated the scaffold performance of a previously described patented stent in a corrosive environment. The patented stent displayed a stronger scaffold performance and more efficiency in reshaping stenotic vessel during degradation. It has been suggested that the scaffold performance of biodegradable stents highly relies on their geometries. Benefiting from a clever design, a biodegradable alloy stent with strong scaffold performance can be an alternative choice in surgery.

A pressure of 2.1 MPa was exerted for expansion of the common stent. In previous studies, Li et al. [21] applied a pressure of 1.9 MPa for stent expansion in 30% stenotic vessels. Because a stenosis ratio of 40% was used in this study, a little higher expansion pressure was applied on both stents. Moreover, Lally et al. [22] applied a pressure of 13 MPa to expand vessels to a diameter greater than the diameter of the stent at the first step of simulation. The expansion pressure of 2.1 MPa was lower than 13 MPa, meaning that it would not damage the stenotic vessel and that it can be accepted in clinical surgery.

Corrosion mechanisms suggest that stress corrosion evolves rapidly during the degradation process [10, 14] and causes severe mass loss at the areas with high stress concentration (Figs. 8, 9). In the case of the patented stent, the clever designs of the strutting ring and the link significantly decreased the maximum stress and stress distribution in struts was uniform. A few elements disappeared in struts of the patented stent even when a crack was generated (Fig. 8). Furthermore, the updated interaction between the strutting ring and the link could continuously prevent the patented stent from recoiling (Fig. 9). Thus, this kind of stent can supply strong support for stenotic vessels although it weakens in a corrosive environment.

6–12 months are required for the remodeling process to be completed [23]. Therefore, biodegradable stents should provide enough support to stenotic vessels for this period of time. However, most biodegradable stents do not meet this requirement due to poor scaffold performance and mass loss. Thus, mass increase and structure optimization are usually targeted to improve the scaffold performance of biodegradable stents [24, 25]. Increasing mass allows the device resisting to uniform corrosion [24] and extended service time. Simultaneously, structural optimization, which contributes to uniform stress distribution on the stent [24, 25], is conductive to inhibit stress corrosion. However, the improvement of scaffold performance using these two conventional methods is limited. In comparison, expectations are greater regarding novel structure design of biodegradable stents. As is shown in Fig. 8, the common stent breaks down at t* = 1. In contrast, only few elements failed in the patented stent, which implies that the patented stent is promised to have a longer service time. Furthermore, optimization design referring to the length, width, thickness and diameter offers better mechanical performance.

Radial recoiling of was small for both stents, even when the crack occurred in the common stent (Fig. 8). The common stent did not completely loose its scaffold ability. Indeed, the other parts of the common stent still afforded the vessel scaffold. In addition, according to the interaction between the stent and the vessel, high stress distributed in the plaque-vessel system may help to stimulate endothelial hyperplasia. Biodegradable stents wrapped with growing vessels are predicted to have a prolonged service time as well as lower risks of thrombosis [26]. This interaction between biodegradable stents and arteries should be explored in further research. In further studies, hemodynamic characteristics induced by stent deployment should be investigated because the wall shear stress (WSS) and von Mises stress affects the endothelial hyperplasia and the reshaping of stenotic vessels.

One limitation is that the vessel wall as an isotropic hyperelastic material whose constitutive relationship is present as a six-parameter Odgen hyperelastic constitutive equation, although the constitutive equation of the vessel varies according to the vessel type [27, 28]. Fetamifar et al. [27] developed the lumen buckling equation for nonlinear anisotropic thick-walled arteries to determine the effect of axial tension based on exponential Fung strain function. Garcia et al. [28] used a two-fiber strain energy density function to characterize the mechanical behavior of veins under torsion. In future research, scaffold effects of the patented stent when it is deployed into stenotic vessels with different types and materials will be investigated.

## Conclusion

In this study, the mechanical performance of the patented stent and its effect on reshaping stenotic vessels upon degradation in a corrosive environment were revealed using the FEA approach. Our results suggest that the patented stent could provide much stronger scaffold for stenotic vessels and have positive influence on reshaping stenotic vessels in a corrosive environment compared with common stents. This implies that structural innovation is very helpful for strong scaffold performance and corrosion resistance. A novel biodegradable zinc alloy stent with sufficient scaffold performance can be a new competitive intervention device for future clinical cardiovascular applications.

## Abbreviations

FEA: finite element analysis
CDM: continuum damage mechanism
SC: stress corrosion
WSS: wall shear stress
